# An Unusual Clinical Presentation of Gastrointestinal Metastasis From Invasive Lobular Carcinoma of Breast

**DOI:** 10.1177/2324709616639723

**Published:** 2016-03-30

**Authors:** Bathmapriya Balakrishnan, Sufiya Shaik, Irina Burman-Solovyeva

**Affiliations:** 1 St Joseph Mercy Ann Arbor, Ypsilanti, MI, USA

**Keywords:** family medicine, lobular breast carcinoma, metastatic disease

## Abstract

*Introduction*. We present an unusual case of metastatic lobular breast carcinoma. Typical areas of metastasis include bone, gynecological organs, peritoneum, retroperitoneum, and gastrointestinal (GI) tract, in order of frequency. With regard to GI metastasis, extrahepatic represents a rare site. *Case*. Two years after being diagnosed with invasive lobular breast carcinoma, a 61-year-old female complained of 3 months of nonspecific abdominal pain and diarrhea. A colonoscopy revealed 5 tubular adenomatous polyps in the ascending and transverse colon. Contrast computed tomography (CT) of the abdomen and pelvis was done 7 months after the colonoscopy to further evaluate persistent diarrhea. The CT results were consistent with infectious or inflammatory enterocolitis. Despite conservative management, symptoms failed to improve and a repeat diagnostic colonoscopy was obtained. Random colonic biopsies revealed metastatic high-grade adenocarcinoma of the colon. *Discussion*. Metastatic lobular breast carcinoma to the GI tract can distort initial interpretation of endoscopic evaluation with lesions mimicking inflammation. The interval between discovery of GI metastasis and diagnosis of lobular breast cancer can vary widely from synchronous to 30 years; however, progression is most often much sooner. Nonspecific symptoms and subtle appearance of metastatic lesions may confound the diagnosis. A high index of suspicion is needed for possible metastatic spread to the GI tract in patients with a history of invasive lobular breast carcinoma. Perhaps, patients with nonspecific GI symptoms should have an endoscopic examination with multiple random biopsies as invasive lobular carcinoma typically mimics macroscopic changes consistent with colitis.

## Clinical Case

In January 2015, a 61-year-old lady presented to her internist with 3 months of dull right lower quadrant abdominal pain and diarrhea. Her symptoms began since completing several courses of antibiotics for urinary tract infection and were aggravated by consumption of dairy products and red meat. She had bowel movements up to 3 times daily with nocturnal fecal incontinence. Her pain was constant, severe, and associated with nighttime awakening without specific triggers. She had lost a total of 10 lbs of weight. Pertinent negatives included recent travel, blood or mucous in stool, new medications, sick contacts, and rashes.

She was diagnosed in June 2012 with invasive lobular carcinoma of the left breast (stage IIIC, T3, pN3a, M0, GX) characterized by strongly positive estrogen receptor (ER) and progesterone receptor (PR) but human epidermal growth factor receptor 2 (HER2-neu) negative. She also had diarrhea-predominant irritable bowel syndrome (IBS), colonic diverticulosis, and well-controlled type 2 diabetes mellitus.

Initial staging of breast malignancy was T3N1 after which she completed 4 cycles of neoadjuvant chemotherapy with docetaxel and cyclophosphamide by October 2012. Following bilateral mastectomy in December 2012, her breast cancer was restaged to pT3N. Radiotherapy to the left breast was completed in April 2013. Her maintenance therapy consisted of anastrozole, but due to side effects, she was swapped to letrozole in May 2013. At follow-up in July 2014, her oncologist discussed with her possible longer term aromatase inhibitor usage beyond the requisite 5 years.

Family history is negative for inflammatory bowel disease (IBD). However, she had one cousin with colon cancer, an uncle with lung cancer, an aunt with breast cancer, and another aunt with stomach cancer. She had a smoking history of 31 pack years and consumed alcohol minimally.

A chronological review of the patient’s gastrointestinal (GI) investigations included the following:

1. In 2008 tests for celiac disease, which were prompted by diarrhea, were negative.
2. Screening colonoscopy in March 2014 with limited preparation to the cecum revealed 5 tubular adenomatous polyps in the ascending and transverse colon with recommendation for a follow-up colonoscopy in 1 year.
3. Blood work in November 2014 showed normal complete blood count (CBC) and normal renal and liver function.
4. Contrast computed tomography (CT) of the abdomen and pelvis obtained in November 2014 during the onset of her GI symptoms revealed mild to moderate wall thickening in the jejunum and in multiple segments of the ileum and the rectum. She had patent mesenteric vessels excluding ischemic colitis. Venous congestion was difficult to exclude; hence, infectious or inflammatory cause for enterocolitis was suspected.

**Figure 1.**
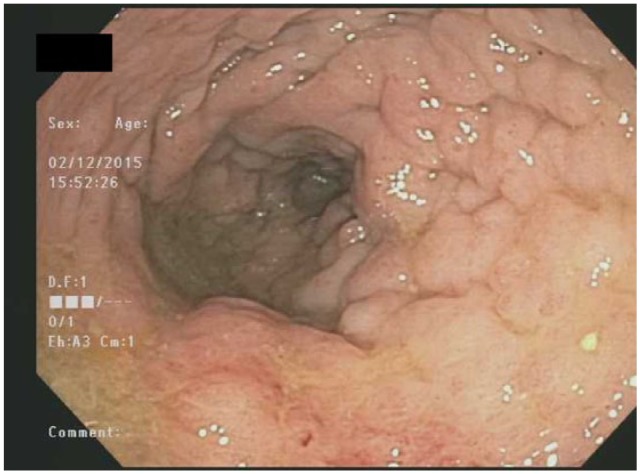
The patient’s rectosigmoid colon as seen during colonoscopy, showing an inflamed appearance frequently seen in IBD.

A gastroenterology review in February 2015 culminated in non-revelatory laboratory testing for inflammatory and infectious diarrhea. The testing included CBC, comprehensive metabolic panel (CMP), C-reactive protein (CRP), and erythrocyte sedimentation rate (ESR). Stool studies of culture and stain, tests for ova and parasites, *Clostridium difficile* toxin A and B enzyme immunoassay (EIA), and *Giardia/Cryptosporidium* antigen detection (GLCRA) tests were sent. An expedited colonoscopy was scheduled for 6 days after her initial appointment. The patient’s rectosigmoid colon showed an inflamed appearance frequently seen in IBD (Figure 1). Biopsy samples, however, revealed metastatic high-grade adenocarcinoma of the colon. Immunohistopathological analysis supported the diagnosis of metastatic GI malignancy from a breast primary (Figure 3).

Although the recommended treatment was palliation and hospice care, the patient opted for life-prolonging therapies. At present, she is enrolled into a phase II clinical trial of fulvestrant (Faslodex) plus mammalian target of rapamycin inhibitor (everolimus) starting in March 2015. Unfortunately, imaging studies in May 2015 revealed progressive bone and liver metastasis.

## Discussion

Annually, approximately 230 000 women and 2300 men are afflicted with a new diagnosis of breast cancer. It is the second most common malignancy occurring in women after skin cancer in the United States.^1^ Historical trends show that since the 2000s, overall incidence of breast cancer is decreasing and has reached a steady state.^1^ The most common subtype of invasive breast carcinoma is ductal followed by lobular carcinoma. Although lobular carcinoma accounts for 5% to 15% of newly diagnosed breast cancers, its incidence is increasing while invasive ductal carcinoma remains stable.^2^ Invasive lobular carcinoma is more prevalent in the older age groups of 57 to 64 years.

Common metastatic sites of breast cancer are the liver, lung, brain, and bones. GI metastatic disease is rare from a primary breast malignancy. Out of 12 001 cases of primary breast cancer, Matsuda et al reported 73 cases of GI spread, of which only 24 cases presented as colorectal metastatic disease.^3^ Less than 1% of GI metastatic disease was noted in 2500 cases of primary breast cancer over an 18-year follow-up period.^4,5^ It is more common to discover a second primary GI cancer in patients with prior history of breast cancer.^6^ Metastatic spread of lobular breast carcinoma preferentially occurs in the bones, gynecological organs, peritoneum, retroperitoneum, and GI tract in comparison to ductal carcinoma, which spreads to the liver, lung, and brain tissues.^2^

**Figure 2.**
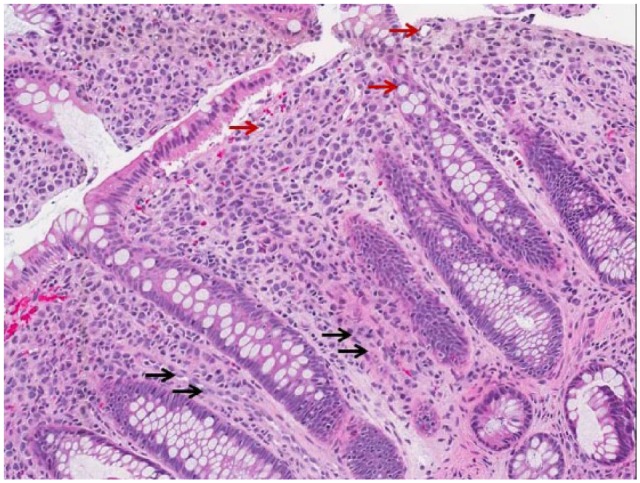
The patient’s colonoscopy biopsy showing histological hallmark of invasive lobular carcinoma with single file arrangement of cells, also known as the “Indian file appearance” (black arrows) and signet-ring morphology (red arrows).

The growth pattern of metastatic lobular breast carcinoma can distort initial interpretation of endoscopic evaluation of the GI tract. Suspicious lesions mimic the appearance of IBD or primary colon cancer,^3^ as shown in Figure 1. Lesions are usually numerous and diffusely infiltrative causing narrowing of large bowel lumen.^6^ Metastatic lobular carcinoma infiltrates within the serosal, muscular, and submucosal layers with cord-like projections of small cells,^4,7^ as can be seen in Figure 2. Endoscopic and radiological appearances are akin to linitis-plastica lesions that have circumferential thickening and stricture of the colorectum.^2,3,8^ When the terminal ileum is affected, it may adopt the radiologic and macroscopic appearance of Crohn’s disease.^3,9,10^ Characteristically in metastatic invasive lobular carcinoma, single file arrangement of tumor cells with signet ring morphology is seen,^2,3^ although this has also been observed in gastric carcinoma.^2^ Immunohistochemical analysis of metastatic invasive lobular carcinoma will exhibit positivity for gross cystic disease fluid protein-15 (GCDFP-15), ER, and PR unlike primary colorectal or gastric carcinoma. However, in 20% to 28% of cases, ER+ cells were noted in primary gastric cancer.^2^ Other potential antigenic markers include CK 7 and CK 20, MUC 1, MUC 2, and GCDFP-15.^6^

**Figure 3.**
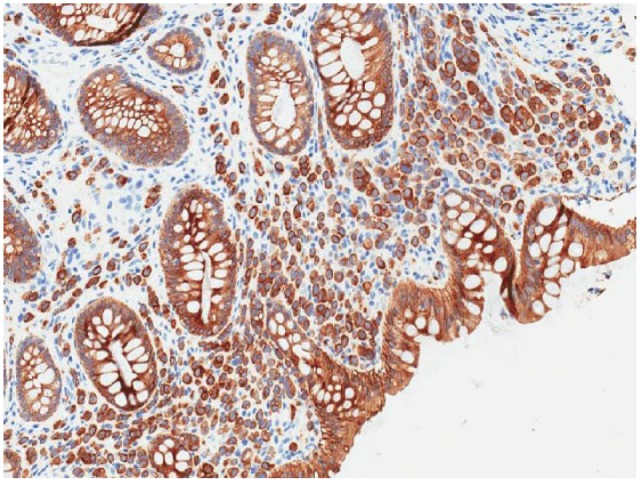
The patient’s colonoscopy biopsy with immunohistochemical cytokeratin staining confirming metastatic lobular breast carcinoma in the GI tract.

The interval of time from diagnosis of breast cancer to the discovery of GI metastatic disease can vary widely from synchronous to 30 years, and occasionally, the discovery of GI metastatic disease precedes breast cancer diagnosis.^2,11,12^ Matsuda et al^3^ and Schwarz et al^11^ reported a median interval of GI metastatic progression of 6 years with a range of 0.25 to 12.5 years. Patients may present with a variety of nonspecific abdominal symptoms including diarrhea, constipation, nausea, dysphagia, anorexia, and hematochezia. They may also be asymptomatic with hemepositive stools.^6,11^ Unfortunately, prognosis for patients with GI metastatic disease from breast primary is poor with only a few patients surviving beyond 2 years.^2^ At presentation, patients usually have widespread metastatic disease and are offered systemic hormonal and/or chemotherapy with surgery.^4^

Nonspecificity of GI symptoms may present a hurdle for referral for invasive or definitive investigation. Frequently, patients go through a multitude of laboratory testing that may be noncontributory, causing false reassurance and hence delay in diagnosis. Recognizing that the time to progression for metastatic GI disease is variable but that the median progression occurs within the first 6 years is important. Also, GI metastatic disease confers poorer prognosis, possibly due to hematogenous spread with cancer seeding in other organ systems compared to usual lymphocytic spread of breast cancer. As the metastatic lesions infiltrate serosal, mucosal, and submucosal layers in a single file pattern, they may be flat or subtle and therefore evade biopsy.

This case highlights the importance of understanding the clinical progression and natural history of a growing subtype of breast cancer. A high index of suspicion for possible GI metastatic disease in patients with a history of invasive lobular breast carcinoma, whether newly diagnosed or in remission, is needed. Perhaps, patients with diarrhea-predominant GI manifestation should have endoscopic examination with multiple random biopsies of colonic mucosa,^13^ as invasive lobular carcinoma typically mimics macroscopic changes seen in IBD.
